# Can the activation of plasminogen/plasmin system of the host by metabolic products of *Dirofilaria immitis* participate in heartworm disease endarteritis?

**DOI:** 10.1186/s13071-015-0799-0

**Published:** 2015-04-01

**Authors:** Javier González-Miguel, Rodrigo Morchón, Elena Carretón, José Alberto Montoya-Alonso, Fernando Simón

**Affiliations:** Laboratory of Parasitology, Faculty of Pharmacy, Institute of Biomedical Research of Salamanca (IBSAL) and University of Salamanca, 37007 Salamanca, Spain; Internal Medicine, Faculty of Veterinary Medicine, University of Las Palmas de Gran Canaria, 35413 Arucas, Las Palmas Spain

**Keywords:** *Dirofilaria immitis*, Excretory/secretory antigens, Fibrinolysis, Plasmin, Endothelial cells, Smooth muscle cells, Proliferative endarteritis

## Abstract

**Background:**

Proliferative endarteritis is one of the key pathological mechanisms of cardiopulmonary dirofilariosis, a cosmopolitan parasitosis caused by *Dirofilaria immitis* affecting dogs and cats around the world. It has been shown that the excretory/secretory antigens from *D. immitis* adult worms (DiES) bind plasminogen (PLG) and activate fibrinolysis, which can lead to a survival mechanism for the parasite in its intravascular environment. However, overproduction of plasmin (final product of the route) has been related to pathological processes similar to those described in proliferative endarteritis. The aim of this study is to relate the appearance of this pathological condition with the activation of the PLG/plasmin system of the host by DiES.

**Methods:**

Cell proliferation through the crystal violet technique, cell migration by wound healing assay and degradation of the extracellular matrix by measuring collagen degradation and levels of matrix metalloproteinases were studied in an “*in vitro*” model using canine vascular endothelial and smooth muscle cells. These cells were treated with a mixture of DiES + PLG. Untreated cells, cells only stimulated with DiES or with PLG, or with a mixture of DiES + PLG + εACA (an inhibitor of the PLG-plasmin conversion) were employed as controls. In addition, the effect of DiES on the expression of the fibrinolytic activators tPA and uPA, the inhibitor PAI-1 and the PLG receptor Annexin A2 was analyzed in both types of cultures by western blot.

**Results:**

Plasmin generated by DiES + PLG binding produced a significant increase in the cell proliferation and migration of the endothelial and smooth muscle cells, as well as an increase in the destruction of the extracellular matrix based on a further degradation of Type I Collagen and an increased level of matrix metalloproteinase-2. DiES also induce an increase in the expression of tPA and uPA in endothelial cells in culture, as well as a decrease in the expression of PAI-1 in both types of cells.

**Conclusions:**

Our study reports an interrelationship between plasmin caused by fibrinolysis activation by metabolic products of *D. immitis* and the appearance of pathological events similar to those described in the emergence of proliferative endarteritis in the cardiopulmonary dirofilariosis.

## Background

*Dirofilaria immitis* is the filaroid nematode that causes the canine and feline cardiopulmonary dirofilariosis, a vector-borne parasitosis with cosmopolitan distribution. *D. immitis* is also responsible for the human pulmonary dirofilariosis, a clinical entity characterized by the formation of benign lung nodules that may be confused with carcinomas in radiology [1].

The dog acts as definitive host and reservoir of the parasite. The adult worms lodge in its pulmonary artery and right ventricle of the heart causing a chronic inflammatory pathology at vascular level [2]. One of the key factors of the disease is the appearance of proliferative endarteritis, which has resulted in the formation of intravascular microvilli. This process has previously been described as being accompanied by an increase and migration of the arterial wall cells [3-6], as well as the destruction of the extracellular matrix (ECM) [7]. These changes cause disorganization of the endothelium and reduction of the vascular lumen in the pulmonary arteries, with the consequent extension of pathology to the pulmonary parenchyma [8]. On the other hand, the simultaneous death of groups of adult worms can trigger an acute disease characterized by the exacerbation of the inflammatory reactions and the emergence of serious thromboembolic events threatening the life of the affected hosts [9]. However, *D. immitis* possesses the ability to regulate these pathological mechanisms and survive for long periods (over 7 years) in their intravascular environment. Recently, it has been shown that both excretory/secretory and surface-associated antigens of *D. immitis* interact with the fibrinolytic system of the host binding PLG and generating plasmin [10,11].

PLG is a glycoprotein predominantly released by the liver into the blood circulation. After its activation PLG becomes a serine protease (plasmin), whose main target are the fibrin clots. Under physiological conditions, this process is strictly regulated at the vascular level for the complex formed by receptors that bind PLG through carboxyterminal lysine residues (Annexin A2, among others) and activators [tissue plasminogen activator (tPA) and urokinase-type plasminogen activator (uPA)], whose activity is inhibited primarily by the plasminogen activator inhibitor-1 (PAI-1) [12,13].

The activation of the fibrinolytic system by *D. immitis* antigens and the consequent maintenance of haemostasis, a priori beneficial for both the parasite and host, could have pathological consequences. An over-activation of the PLG/plasmin system has been related to cell invasion and intra-organic migration of different pathogens [14,15]. In addition, in human cardiovascular research the overproduction of plasmin has also been linked with the proliferation and migration of human vascular cells and with the degradation of extracitoplasmatic matrices [16-19]. These mechanisms have similarities with those that cause the formation of microvilli in the cardiopulmonary dirofilariosis, although their molecular aspects have not been conveniently studied to date in this parasitosis.

The objective of this work is to demonstrate that the overproduction of plasmin by the antigens of *D. immitis* may relate to the mechanisms described in the formation of microvilli at the vascular level in cardiopulmonary dirofilariosis. For this purpose, cell proliferation and migration, degradation of the ECM and expression of some components of the fibrinolytic system were studied in canine vascular endothelial and smooth muscle cells in culture stimulated with the parasitic antigens and PLG.

## Methods

### Cell culture

Canine endothelial cells (CnAOEC) and canine smooth muscle cells (CnAOSMC) from Cell Applications, INC were respectively grown in canine endothelial and canine smooth muscle cell growth mediums (Cell Applications, INC). CnAOEC plates were precoated with an attachment factor solution (Cell Applications, INC). Cells were cultured at 37°C in a humidified atmosphere in the presence of 5% carbon dioxide and 95% air. Medium was changed every 3 days. Expansion was carried out by trypsinizing the cells, (Trypsin/EDTA, Cell Applications, INC), and re-plating them when the proliferating cells had reached a sufficient density. Passaging was performed at ratios of 1:6 (CnAOEC) or 1:3 (CnAOSMC). Cell counts were performed using the equipment Countess® Automated Cell Counter (Invitrogen) following the manufacturer’s instructions.

### Reagents and stimulation of CnAOEC and CnAOSMC

DiES were prepared as previously described [12] with minor modifications and stored at −80°C. In brief, live worms (25) obtained from a naturally infected dog were washed in sterile phosphate-buffered saline solution (PBS) pH 7.2 and incubated for 24 h in 50 ml of Eagle’s minimum essential medium (EMEM) supplemented with 50 U/ml penicillin and 50 μg/ml streptomycin at 37°C. A cocktail of protease inhibitors was added to the medium following the methodology described by Maizels *et al*. [20]. The medium was dialyzed against water for 24 h and filtered through an Amicon YC05 membrane (Millipore). The protein concentration of DiES was measured by DC protein assay commercial kit (Bio-Rad). DiES extract was tested for the presence of endotoxin contamination using a quantitative *Limulus* amebocyte lysate test (BioWhittaker). The endotoxin quantity was under the sensitivity level of cell stimulation (<0.4 U/mg protein).

For stimulations, CnAOEC and CnAOSMC were grown for 4 days to obtain confluent cultures and were treated with 1 μg/ml of DiES [21], 10 μg/ml of PLG (Acris Antibodies) [17] or with a mixture of both treatments. Untreated cells and cells treated with DiES + PLG + 50 mM of the lysine analogue ε-aminocaproic acid (εACA) as an inhibitor of PLG activation were used as control cells under the same conditions.

### Cell proliferation assay

Cells were plated on 24-well plates to a density of 10^4^ CnAOEC/well or 1.5 × 10^4^ CnAOSMC/well and allowed to attach overnight. After stimulation cell proliferation was analyzed by crystal violet nuclei staining over 10 days to determine the number of viable cells, as previously described [22]. Briefly, every two days, cells were rinsed with PBS, fixed with 4% formaldehyde for 10 min and stained with 0.2% crystal violet for 30 min at room temperature. After several rinses with PBS, the cells were allowed to dry overnight and crystal violet bound to cells was extracted by incubation with 2 ml/well of 10% acetic acid. The absorbance of the samples was then measured at 595 nm and transformed to “number of viable cells” using a curve that correlated absorbance and number of endothelial or smooth muscle cells previously determined.

### Cell migration assay

Cell migration was assessed by quantifying the percentage of wound closure in the wound-healing assay [23]. In brief, CnAOEC and CnAOSMC were cultured in 60 mm plates (3 × 10^5^ cells/plate) and allowed to attach overnight before wound creation. Confluent monolayers were wounded using a sterile pipette tip and the medium was exchanged for fresh medium before cell stimulation to remove cellular debris. The extent of wound closure of the treated and control cells was monitored over a time course by microscopy and determined along 8 hours by calculating the migrated distance/total wound distance.

### Collagen degradation assay

The concentration of collagen in the supernatants was analyzed by ELISA. Treated and control cells were cultured with medium for 48 hours. Then the culture supernatants were collected, filtered and added to multi-well plates (Costar). After incubation overnight at 4°C, wells were blocked with 1% BSA in PBS and incubated with a rabbit anti-Type I Collagen antibody (1:2500) (Acris Antibodies), followed by a peroxidase-conjugated goat anti-rabbit IgG (Sigma) at 1:500 dilution. All incubations were performed for 1 h at 37°C and between each step washed three times with PBS wash buffer (PBS containing 0.05% Tween_20_). Ortho-phenylene-diamine was used as a chromogen. Optical densities (OD) were measured at 492 nm in an Easy Reader (Bio-Rad).

### Matrix metalloproteinases (MMPs) levels assays

The levels of the MMP-2 and MMP-9 metalloproteinases in the culture media of the different experimental groups were analyzed by gelatin zymography according to the methodology described by Marangoni *et al*. [24]. Media samples employed in the collagen degradation assays were electrophoresed on a 10% polyacrylamide gel copolymerized with 1% gelatin (Sigma) together with a MMP marker (Cosmobio) as a positive control. The gels were washed for 1 hour in 2.5% Triton X-100 and incubated for 20 hours at 37°C in agitation in an enzymatic activation buffer ph 7.5 (50 mM Tris; 200 mM NaCl; 5 mM CaCl2; 0,2% Brij-35). The gels were finally stained with Coomassie blue. The positivity was assessed as appearance of clear bands on a dark background with molecular weights of 72 kDa (MMP-2) and 92 kDa (MMP-9). The levels of the MMPs were calculated after measuring the density of the existing bands, which is directly proportional to the amount of gelatin degraded into the gel.

### Cell lysates and Western blot analyses

Western blot analyses were performed as previously described [21] with slight modifications. CnAOEC and CnAOSMC previously treated with 1 μg/ml of DiES for 24 h were lysed in ice-cold lysis buffer (20 mM Tris–HCl (pH 7.5), 140 mM NaCl, 10 mM ethylendiaminetetraacetic acid, 10% glycerol, 1% Igepal CA-630, aprotinin, pepstatin, and leupeptin at 1 μg/ml each, 1 mM phenylmethylsulfonyl fluoride, and 1 mM sodium orthovanadate). Non-stimulated cells were used as controls under the same conditions. Protein samples (10 μg) were separated by SDS-PAGE under reducing conditions and blotted onto polyvinylidine difluoride membranes. Membranes were blocked before incubation with primary antibodies: anti-tPA, anti-uPA, anti-Annexin A2 and anti-PAI-1 (Santa Cruz Biotechnology Inc) according to the manufacturer’s recommendations. After incubation with HRP-conjugated secondary antibodies, bands were visualized by a luminol-based detection system with p-iodophenol enhancement. Anti-α-tubulin antibody (Oncogene Research Products) was used to confirm loading of comparable amount of protein in each lane. Protein expression was quantified by densitometry using Scion Image Software (Scion).

### Statistical analysis

Cell proliferation and migration, collagen degradation and MMP levels significance measurements (comparisons between groups) were performed by ANOVA and corrected for repeated measurements when appropriate. If ANOVA revealed overall significant differences, individual means were evaluated post hoc using Bonferroni’s procedure. The results from the Western blots for the tPA, uPA, annexin A2 and PAI-1 expression were analyzed with the Student’s *t*-test. All the results were expressed as the mean ± SD of three experiments performed with duplicates. In all experiments, a significant difference was defined as a p-value of <0.05 for a confidence level of 95%.

## Results

### DiES produce proliferation of CnAOEC and CnAOSMC via PLG/plasmin system

**Figure 1:**
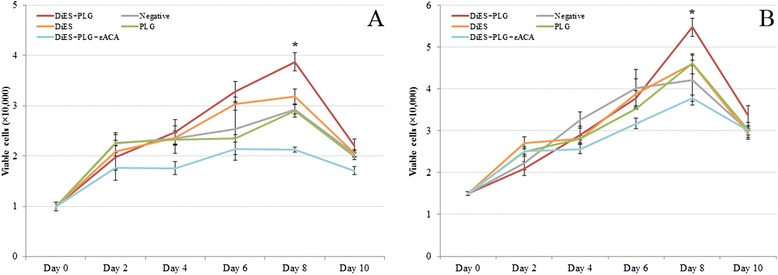
**Cell proliferation assay performed by the crystal violet technique measuring cell viability over a 10 day period.** The experiment was carried out in canine endothelial **(A)** and smooth muscle cells **(B)** untreated or treated with 1 μg/ml of DiES + 10 μg/ml of PLG, 1 μg/ml of DiES, 10 μg/ml of PLG, or with 1 μg/ml of DiES + 10 μg/ml of PLG + 50 mM of the εACA. Results are expressed as number of viable cells (x 10,000). Each point is the mean ± SD from three independent experiments. The asterisk (*) designates significant (p < 0.05) differences between DiES + PLG treatment and control groups.

The effect of DiES and PLG on the proliferation of endothelial and smooth muscle cells was quantified using the crystal violet technique in a period of 10 days (Figure 1). Both cultures showed typical curves of cell growth in all experimental groups with a progressive growth between days 0 and 8 post-treatment, experiencing cell death and an evident decrease of viable cells between days 8 and 10 post-treatment. Crystal violet staining showed an increase significantly greater in the number of viable cells in CnAOEC and CnAOSMC in culture stimulated with DiES + PLG than that showed by other experimental groups on day 8 post-treatment (p < 0.05), indicating that this treatment stimulates the proliferation of CnAOEC and CnAOSMC.

### DiES produce migration of CnAOEC and CnAOSMC via PLG/plasmin system

**Figure 2:**
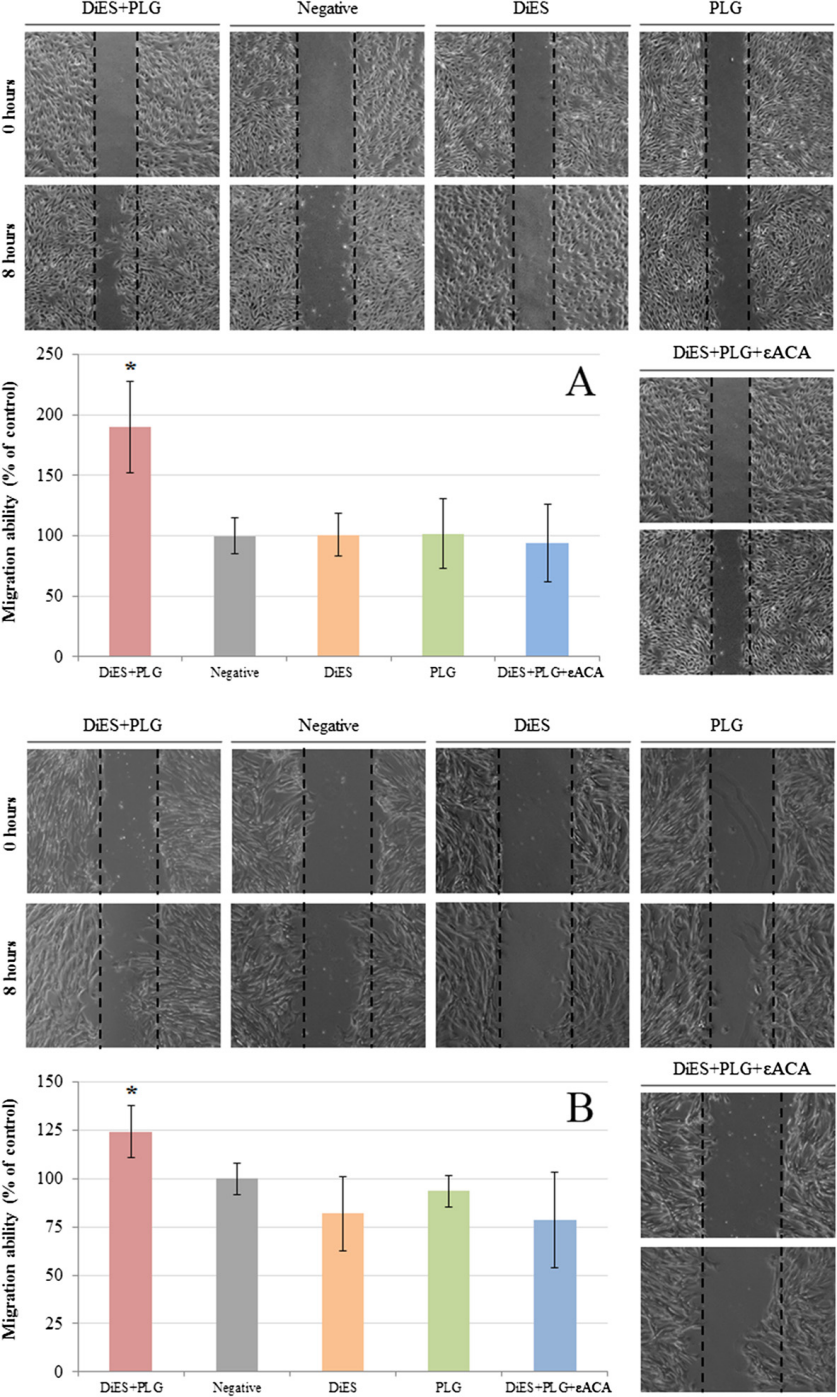
**Cell migration by wound-healing assay.** Confluent cell cultures were wounded post-treatment and migration distances were measured at 8 hours. The experiment was carried out in canine endothelial **(A)** and smooth muscle cells **(B)** untreated or treated with 1 μg/ml of DiES + 10 μg/ml of PLG, 1 μg/ml of DiES, 10 μg/ml of PLG, or with 1 μg/ml of DiES + 10 μg/ml of PLG + 50 mM of the εACA. The results were expressed as percentage of the migration ability of the negative control cells (100%). Each point is the mean ± SD from three independent experiments. The asterisk (*) designates significant (p < 0.05) differences between DiES + PLG treatment and control groups.

A Wound Healing assay was performed to assess migration of endothelial (Figure 2A) and smooth muscle cells (Figure 2B). The quantification was carried out by measuring the distance of migration in comparison with negative control (untreated cells) up to 8 hours post-treatment. In both CnAOEC and CnAOSMC cultures a significant increase of cell migration after stimulation with DiES + PLG with respect to the other experimental groups (p < 0.05) occurred, this increase was most pronounced in cultured endothelial cells.

### DiES produce ECM degradation of CnAOEC and CnAOSMC via PLG/plasmin system

**Figure 3:**
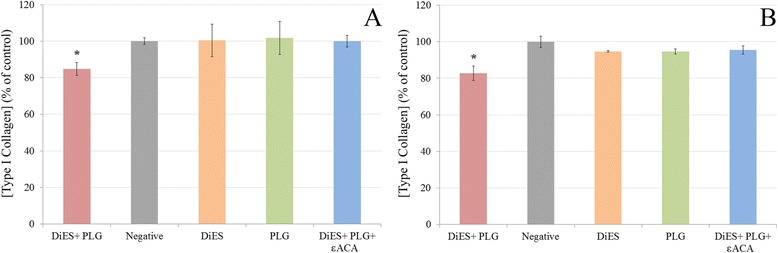
**Type I Collagen degradation assay measured in culture supernatants from canine endothelial (A) and smooth muscle cells (B) untreated or treated with 1 μg/ml of DiES + 10 μg/ml of PLG, 1 μg/ml of DiES, 10 μg/ml of PLG, or with 1 μg/ml of DiES + 10 μg/ml of PLG + 50 mM of the εACA.** The results were expressed as percentage of the Type I Collagen concentration in the culture supernatant from negative control cells (100%). Each point is the mean ± SD from three independent experiments. The asterisk (*) designates significant (p < 0.05) differences between DiES + PLG treatment and control groups.

**Figure 4:**
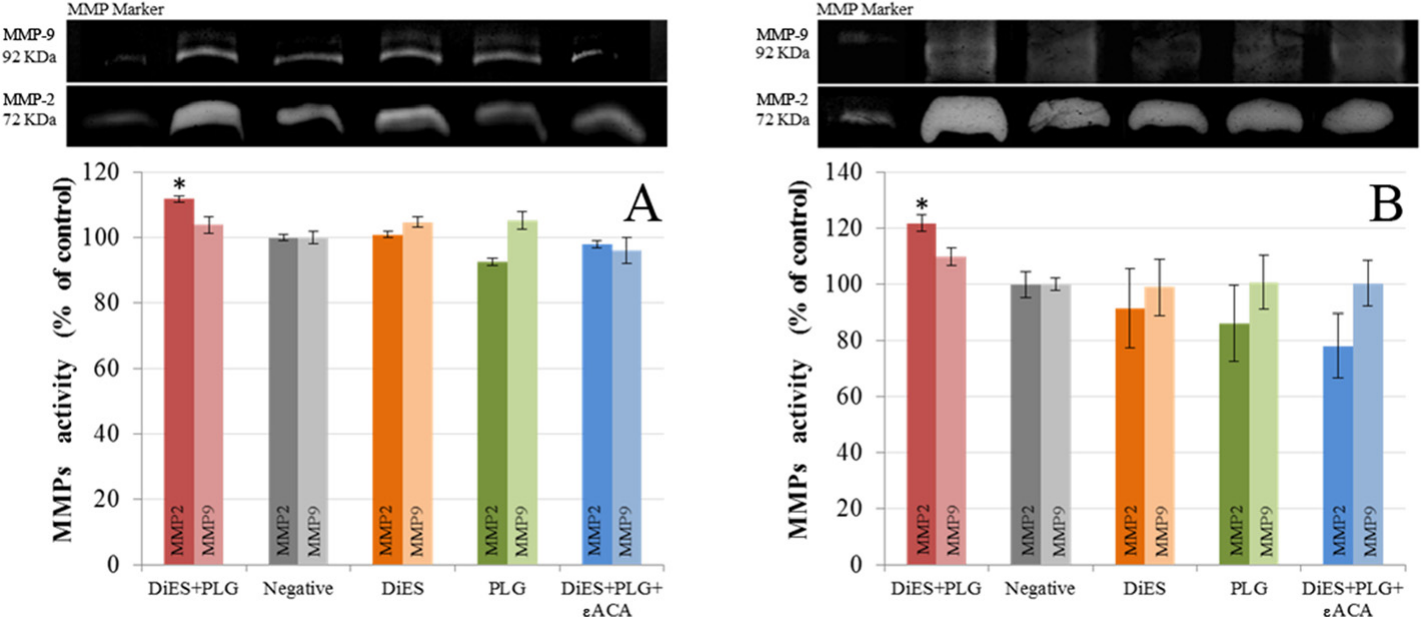
**Representative zymography of culture supernatants from canine endothelial (A) and smooth muscle cells (B) untreated or treated with 1 μg/ml of DiES + 10 μg/ml of PLG, 1 μg/ml of DiES, 10 μg/ml of PLG, or with 1 μg/ml of DiES + 10 μg/ml of PLG + 50 mM of the εACA.** Note the gelatinolytic bands associated with MMP-2 (72 KDa) and MMP-9 (92 KDa) levels. The results are expressed as percentage of the MMPs levels in the culture supernatant from negative control cells (100%). Each point is the mean ± SD from three independent experiments. The asterisk (*) designates significant (p < 0.05) differences between DiES + PLG treatment and control groups.

To examine ECM degradation, Type I Collagen in the culture supernatant of treated and untreated CnAOEC and CnAOSMC were measured by ELISA (Figure 3). A lower concentration of Type I Collagen and therefore a further degradation of the secreted collagen by the cells was observed in the CnAOEC and CnAOSMC stimulated with DiES + PLG than that obtained by the control cells (p < 0.05). In addition, the same culture media from treated and untreated cells was analyzed with gelatin zymography for MMP-2 and MMP-9 levels (Figure 4). Density of the bands was measured by the Quantity One Software (Bio-Rad). The results show a significantly higher level of MMP-2 in the CnAOEC and CnAOSMC treated with DiES + PLG than that obtained in the control cells (p < 0.05). No significant differences in the MMP-9 levels were observed (Figure 4).

### Effect of DiES on the fibrinolytic system components (tPA, uPA, Annexin II and PAI-1) expression in CnAOEC and CnAOSMC

**Figure 5:**
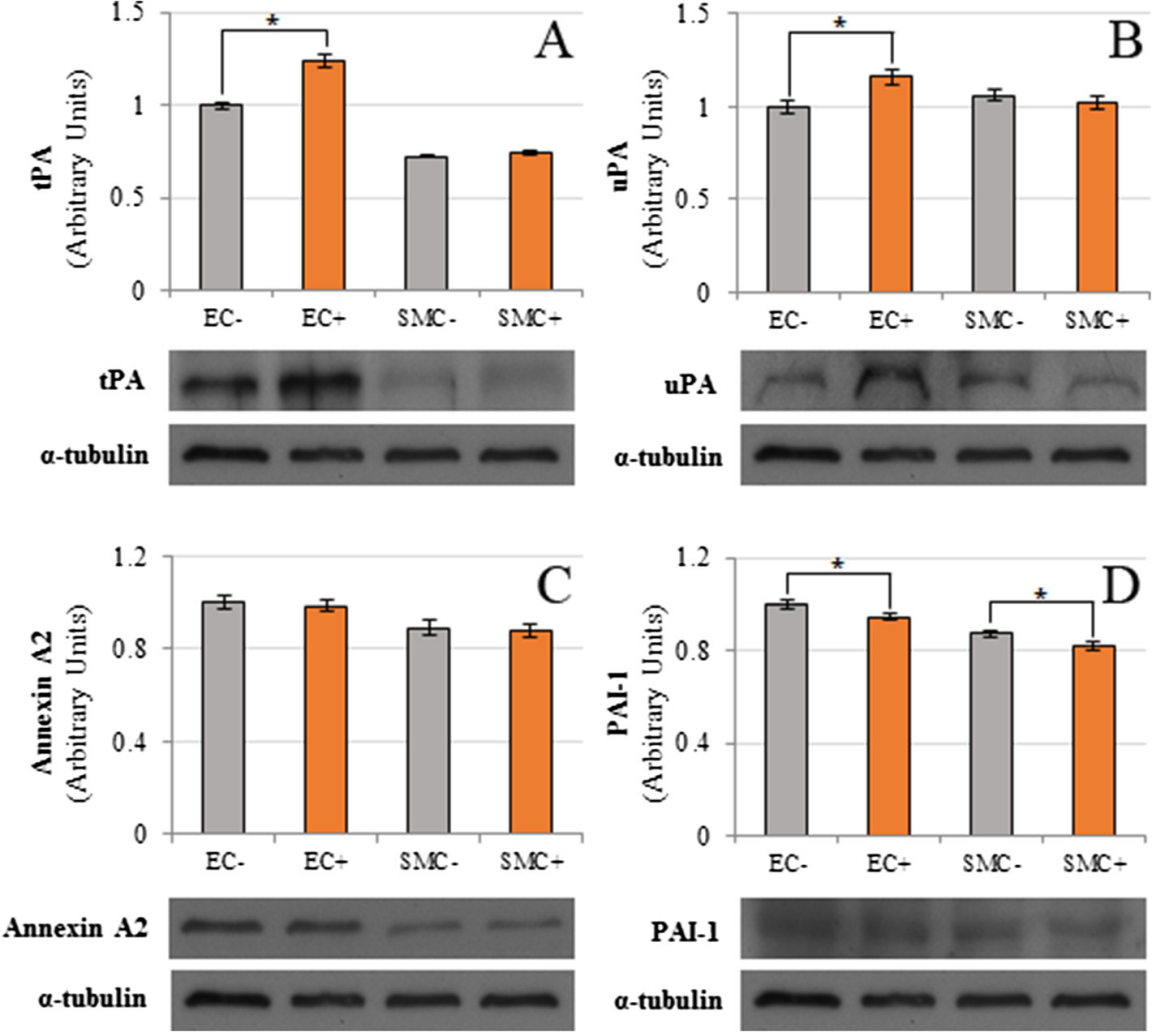
**Effect of DiES on the expression of tPA (A), uPA (B), annexin A2 (C) and PAI-1 (D) in canine vascular endothelial (EC) and smooth muscle cells (SMC).** Protein extracts from lysed DiES untreated or treated confluent cell cultures were analyzed by Western blot for tPA, uPA, annexin A2 and PAI-1. α-tubulin served as a protein control. Results were expressed as the mean ± SD of at least 3 independent experiments. The asterisk (*) designates significant (p < 0.05) differences from control cells. (Gray bars) Non-treated control cells. (Orange bars) Stimulated endothelial or smooth muscle cells with 1 μg/ml of DiES.

To determine the effect of DiES on some of the main components of the fibrinolytic system, proteins from DiES-treated or untreated CnAOEC and CnAOSMC extracts were separated by SDS–PAGE and analyzed by Western blotting using anti-tPA, anti-uPA, anti-Annexin A2 and anti-PAI-1 antibodies. DiES induced a marked increase in the expression of the main fibrinolytic activators tPA and uPA in cultured endothelial cells (p<0.05) (Figure 5A and B), as well as a slight decrease in the expression of the main fibrinolytic inhibitor PAI-1 in both types of cultures (p<0.05) (Figure 5D). Significant differences in the expression of tPA and uPA in CnAOSMC (Figure 5A and B) and in the expression of Annexin A2 in both cell types between DiES-treated or untreated cultures were not found (Figure 5C).

## Discussion

Plasmin is the serine protease responsible for initiating the fibrinolytic process through the lysis of the fibrin clots. Apart from its pivotal role in the maintenance of haemostasis, plasmin possesses exceptionally broad specificity for target substrates playing important physiological and pathological roles in tissue remodeling, cell migration, wound healing, angiogenesis, inflammation and degradation of ECM [25]. In addition, plasmin is also upregulated in chronic inflammatory diseases, including atherosclerosis and arthritis [26]. The cardiopulmonary dirofilariosis is a chronic disease with an important inflammatory component at the vascular level in which the interaction between the excretory/secretory and surface antigens of its aetiologic agent (*D. immitis*) and the overproduction of plasmin has been recently shown [10,11]. The objective of this work was to establish a relationship between this interaction and the mechanisms responsible for the formation of intravascular microvilli in the cardiopulmonary dirofilariosis. Similar lesions have been previously explained through mechanical damage caused by the sole presence of the adult worms in the pulmonary arteries [27]. However, the pathogenic mechanisms causing the proliferative endarteritis at the molecular level have not been described in cardiopulmonary dirofilariosis.

Firstly, we have developed an “*in vitro*” model of canine endothelial and smooth muscle cells to study the relationship between the excretory/secretory antigens of the parasite and the different cell types of the canine arterial wall. Our data show that stimulation of both types of cultures with DiES + PLG cause the proliferation and migration of CnAOEC and CnAOSMC. Cells treated separately with DiES or PLG did not generate growth curves or migration distances significantly different from non-treated cells. In this sense, Morchón *et al*. [22] observed that the stimulation of human endothelial cells in culture with somatic antigens of *D. immitis* (DiSA) did not alter cell proliferation. Finally, the inhibition of both effects by including the 50 mM εACA in the stimulations demonstrates the participation of plasmin generated by DiES + PLG binding on the proliferation and migration of CnAOEC and CnAOSMC.

Two facts reveal the involvement of plasmin generated by DiES + PLG binding in the degradation of the ECM. First, this treatment for 48 h causes a significant increase in the degradation of the Type I Collagen in the culture media of CnAOEC and CnAOSMC. This molecule represents the main component of the ECM of elastic arteries. Its alteration has been associated with vascular disease and its degradation products with the proliferation and migration of smooth muscle cells in remodelling arteries [28]. These results are consistent with that observed *in vivo* by Wang *et al*. [7] who reported a significantly lower amount of collagen in heartworm-infected dogs than in clinically normal dogs. The degradation of the ECM is also related to the increase in the levels of MMPs. Our results show a significantly higher level of MMP-2 or gelatinase A in the culture media of CnAOEC and CnAOSMC treated with DiES + PLG for 48 h, than that obtained from the other control groups. The MMP-2 can digest a large number of the ECM molecules including Type I, II, III, IV, V and XI collagens, laminin, aggrecan core protein, etc. [29]. In addition, the pathophysiological study of the action of gelatinases shows that an increase in its activity can be responsible for tissue remodeling, hypertrophy, angiogenesis and chronic inflammation [30].

All these data are consistent with previous studies, which relate the over-activation of the PLG/plasmin system with different processes of cell proliferation and migration, as well as the degradation of the ECM [16-19]. However, none of them linked these pathogenic processes with the plasmin generated through the interaction of a blood-borne pathogen with its host fibrinolytic system. To complete the knowledge of this interaction we analyze the participation of DiES in the basal production of the main components of the fibrinolytic system secreted by the cells of the arterial wall. Our results show that DiES increases the expression of the fibrinolytic activators tPA and uPA in CnAOEC during the first 24 h of stimulation, an effect that is not observed in CnAOSMC cultures. These data are consistent with those obtained previously where an increase in the basal production of tPA in human endothelial cells in culture was demonstrated [10]. In addition, the presence of tPA is required for the activation of PLG in various parasites, including *D. immitis* [31]. The increase in the expression of uPA could have special significance since, in addition to its function as an activator of fibrinolysis, it plays a key role in tissue remodeling inducing cell proliferation and migration [32], and high levels in its expression are related to cardiovascular disease [33]. DiES causes a significant reduction in the production of PAI-1 in CnAOEC and CnAOSMC in culture. PAI-1 is a member of the serine protease inhibitor (SERPIN) superfamily and is the primary physiological inhibitor of the tPA and uPA activity [34]. Finally, DiES does not cause significant changes in the expression of Annexin A2 PLG receptor. However, it has recently been shown that *D. immitis* is able to excrete numerous antigens which can carry out similar functions as PLG-binding proteins [10].

## Conclusions

In conclusion, the data obtained in this study suggest that metabolic products of *D. immitis* may tip the fibrinolytic balance towards the generation of plasmin, and that this fact is related to the appearance of pathological phenomena similar to those described in the formation of intravascular microvilli in cardiopulmonary dirofilariosis.

## Abbreviations

DiES: Excretory/secretory antigens from *D. immitis* adult worms
PLG: Plasminogen
ECM: Extracellular matrix
tPA: Tissue plasminogen activator
uPA: Urokinase-type plasminogen activator
PAI-1: Plasminogen activator inhibitor-1
CnAOEC: Canine aortic endothelial cells
CnAOSMC: Canine aortic smooth muscle cells
εACA: Lysine analogue ε-aminocaproic acid
OP: Optical density
MMPs: Matrix metalloproteinases

## References

[1] Simón F, Siles-Lucas M, Morchón R, González-Miguel J, Mellado I, Carretón E (2012). Human and animal dirofilariasis: the emergence of a zoonotic mosaic. Clin Microbiol Rev.

[2] Venco L, Genchi C, Simón F, Simón F, Genchi C, Venco L, Montoya MN (2011). La filariosis cardiopulmonar (*Dirofilaria immitis*) en el perro. La filariosis en las especies domésticas y en el hombre.

[3] Adcock JL (1961). Pulmonary arterial lesions in canine dirofilariasis. Am J Vet Res.

[4] Atwell RB, Sutton RH, Buoro IB (1986). Early pulmonary lesions caused by dead *Dirofilaria immitis* in dogs exposed to homologous antigens. Br J Exp Pathol.

[5] Hidaka Y, Hagio M, Horii Y, Murakami T, Naganobu K, Miyamoto T (2004). Histopathological and Enzyme Histochemical Observations on Mast Cells in Pulmonary Arterial Lesion of Dogs with *Dirofilaria immitis* Infestation. J Vet Med Sci.

[6] Kawabata A, Nakagaki K, Yoshida M, Shirota K (2008). Histopathological comparison of pulmonary artery lesions between raccoon dogs (*Nyctereutes procyonoides*) and domestic dogs experimentally infected with *Dirofilaria immitis*. J Vet Med Sci.

[7] Wang JS, Tung KC, Huang CC, Lai CH (2005). Alteration of extracellular collagen matrix in the myocardium of canines infected with *Dirofilaria immitis*. Vet Parasitol.

[8] McCall JW, Genchi C, Kramer LH, Guerrero J, Venco L (2008). Heartworm disease in animals and humans. Adv Parasitol.

[9] Venco L, Genchi C, Ronaldi L, Cringoli G (2007). Heartworm (*Dirofilaria immitis*) disease in dogs. *Dirofilaria immitis* and *D. repens* in Dog and Cat and Human Infections.

[10] González-Miguel J, Morchón R, Mellado I, Carretón E, Montoya-Alonso JA, Simón F (2012). Excretory/secretory antigens from *Dirofilaria immitis* adult worms interact with the host fibrinolytic system involving the vascular endothelium. Mol Biochem Parasitol.

[11] González-Miguel J, Morchón R, Carretón E, Montoya-Alonso JA, Simón F (2013). Surface associated antigens of *Dirofilaria immitis* adult worms activate the host fibrinolytic system. Vet Parasitol.

[12] Miyashita C, Wenzel E, Heiden M (1988). Plasminogen: a brief introduction into its biochemistry and function. Haemostasis.

[13] Cesarman-Maus G, Hajjar KA (2005). Molecular mechanisms of fibrinolysis. Br J Haematol.

[14] Jong AY, Chen SH, Stins MF, Kim KS, Tuan TL, Huang SH (2003). Binding of *Candida albicans* enolase to plasmin(ogen) results in enhanced invasion of human brain microvascular endothelial cells. J Med Microbiol.

[15] Bernal D, de la Rubia JE, Carrasco-Abad AM, Toledo R, Mas-Coma S, Marcilla A (2004). Identification of enolase as a plasminogen-binding protein in excretory-secretory products of Fasciola hepatica. FEBS Lett.

[16] Nicholl SM, Roztocil E, Galaria II, Davies MG (2005). Plasmin induces smooth muscle cell proliferation. J Surg Res.

[17] Yang Z, Eton D, Zheng F, Livingstone AS, Yu H (2005). Effect of tissue plasminogen activator on vascular smooth muscle cells. J Vasc Surg.

[18] Roth D, Piekarek M, Paulsson M, Christ H, Bloch W, Krieg T (2006). Plasmin modulates vascular endothelial growth factor-A-mediated angiogenesis during wound repair. Am J Pathol.

[19] Hayashi M, Matsuzaki Y, Shimonaka M (2009). Impact of plasminogen on an in vitro wound healing model based on a perfusion cell culture system. Mol Cell Biochem.

[20] Maizels RM, Blaxter ML, Robertson BD, Selkirk ME (1991). Parasite antigen parasite genes: a laboratory manual for molecular parasitology.

[21] Morchón R, González-Miguel J, Mellado I, Velasco S, Rodríguez-Barbero A, Simón F (2010). Adult *Dirofilaria immitis* excretory/secretory antigens upregulate the production of prostaglandin E2 and downregulate monocyte transmigration in an “in vitro” model of vascular endothelial cell cultures. Vet Parasitol.

[22] Morchón R, Rodríguez-Barbero A, Velasco S, López-Belmonte J, Simón F (2008). Vascular endothelial cell activation by adult *Dirofilaria immitis* antigens. Parasitol Int.

[23] Yamaguchi M, Ebihara N, Shima N, Kimoto M, Funaki T, Yokoo S (2011). Adhesion, migration, and proliferation of cultured human corneal endothelial cells by laminin-5. Invest Ophthalmol Vis Sci.

[24] Marangoni NR, Melo GD, Moraes OC, Souza MS, Perri SH, Machado GF (2011). Levels of matrix metalloproteinase-2 and metalloproteinase-9 in the cerebrospinal fluid of dogs with visceral leishmaniasis. Parasite Immunol.

[25] Law RH, Abu-Ssaydeh D, Whisstock JC (2013). New insights into the structure and function of the plasminogen/plasmin system. Curr Opin Struct Biol.

[26] Syrovets T, Simmet T (2004). Novel aspects and new roles for the serine protease plasmin. Cell Mol Life Sci.

[27] Atwell RB, Buoro IB, Sutton RH (1985). Experimental production of lesions in canine pulmonary arteries similar to those produced by *Dirofilaria immitis* infection. Vet Rec.

[28] Bou-Gharios G, Ponticos M, Rajkumar V, Abraham D (2004). Extra-cellular matrix in vascular networks. Cell Prolif.

[29] Nagase H, Visse R, Murphy G (2006). Structure and function of matrix metalloproteinases and TIMPs. Cardiovasc Res.

[30] Klein T, Bischoff R (2011). Physiology and pathophysiology of matrix metalloproteases. Amino Acids.

[31] Figuera L, Gómez-Arreaza A, Avilán L (2013). Parasitism in optima forma: Exploiting the host fibrinolytic system for invasion. Acta Trop.

[32] Nicholl SM, Roztocil E, Davies MG (2005). Urokinase-induced smooth muscle cell responses require distinct signaling pathways: a role for the epidermal growth factor receptor. J Vasc Surg.

[33] Fuhrman B (2012). The urokinase system in the pathogenesis of atherosclerosis. Atherosclerosis.

[34] Iwaki T, Urano T, Umemura K (2012). PAI-1, progress in understanding the clinical problem and its aetiology. Br J Haematol.

